# Histopathological findings of failed blebs after microinvasive bleb surgery with the XEN Gel Stent and Preserflo MicroShunt

**DOI:** 10.1007/s00417-024-06479-w

**Published:** 2024-04-16

**Authors:** Jonas Neubauer, Daniela Suesskind, Caroline J. Gassel, Emil Nasyrov, Bogomil Voykov

**Affiliations:** Department of Ophthalmology, University Eye Hospital, Eberhard Karls University, Elfriede-Aulhorn Str. 7, 72076 Tübingen, Germany

**Keywords:** Bleb fibrosis, Microinvasive glaucoma surgery, Microinvasive bleb surgery, Filtering surgery, Histology, Gel stent, Microshunt

## Abstract

**Purpose:**

The success of XEN Gel Stent (XEN) and Preserflo MicroShunt (Preserflo) implantation depends mainly on the development of bleb fibrosis. This study aimed to describe the histological findings of bleb fibrosis after XEN and Preserflo surgery.

**Methods:**

This retrospective study included patients with different types of glaucoma who underwent revision surgery after XEN or Preserflo implantation. The available clinical information and histological samples of removed fibrotic tissue were analyzed.

**Results:**

Thirty-six patients were included. Revision surgery was performed at a median of 195 (range = 31–1264) days after primary surgery. The mean intraocular pressure changed from 29.1 (± 10.3) mmHg at baseline to 18.3 (± 8.7) mmHg (− 37%; *p* < 0.0001) and 16.2 (± 4.2) mmHg (− 45%; *p* < 0.0001) after 6 and 12 months, respectively. Histological analysis revealed an increase in activated fibroblasts and macrophages in all specimens and a parallel orientation of fibroblasts in a minor part of the probe in 60% of the specimens. No pronounced inflammatory reaction in the form of lymphocytic or granulocytic infiltration was observed. The comparison of specimens from uveitic glaucoma and primary open-angle glaucoma patients revealed no significant differences.

**Conclusions:**

The histological analysis of fibrotic blebs from the XEN and Preserflo implants did not show any pronounced immune or foreign-body reaction and revealed a similar histological pattern of failed blebs after trabeculectomy.

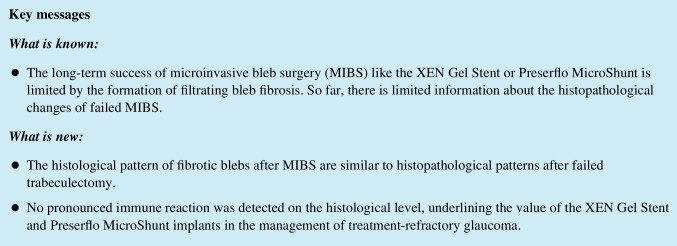



## Introduction

Glaucoma is one of the most common causes of blindness worldwide and results in loss of retinal ganglion cells, eventually leading to relative and absolute scotomas and vision loss, if the condition is left untreated. In patients where topical medication proves insufficient in achieving the desired level of IOP control, surgical intervention may become a necessary consideration. Trabeculectomy is the current gold standard of glaucoma surgery, which can result in long-lasting reduction of intraocular pressure (IOP). However, there are known side effects and complications, such as persistent hypotony, bleb-associated endophthalmitis, and bleb failure due to fibrosis [1–3]. In recent years, there has been a shift toward microinvasive glaucoma surgery in an attempt to reduce the risks of glaucoma surgery [4]. Microinvasive bleb surgery (MIBS) with the XEN Gel Stent (XEN) and Preserflo MicroShunt (Preserflo) has shown promising results in different types of glaucoma [5–11]. While a retrospective study has reported comparable results for microshunt and trabeculectomy, a recent prospective study has shown that trabeculectomy has a higher surgical success rate, although it requires more postoperative interventions. Therefore, the superiority of one procedure over the other remains a topic of ongoing debate [12, 13]. Similar to trabeculectomy, the success of MIBS is limited by the formation of postoperative bleb fibrosis, which can lead to elevated IOP and, ultimately, treatment failure [14]. However, fibrosis is observed in up to 45% of patients even when using the antimetabolite Mitomycin-C (MMC) [15]. Currently, there are limited data about the histopathological alteration of blebs after MIBS. Therefore, a better understanding of the pathophysiology of bleb fibrosis after MIBS may help further improve surgical success.

This study thus aimed to investigate the histopathological findings of failed blebs after MIBS with the XEN or Preserflo.

## Methods

This retrospective study analyzed the data of patients who underwent surgical bleb revision after XEN or Preserflo implantation from 01/01/2015 to 06/30/2022 at the University Eye Hospital Tuebingen, Germany. The study included patients with different types of glaucoma who had at least 1 month of follow-up. Incisional or laser surgery before XEN or Preserflo implantation was not an exclusion criterion. All MIBS and surgical bleb revision surgery were performed by one experienced glaucoma surgeon (BV). Briefly, the conjunctiva was opened at the limbus with two radial incisions. The conjunctiva was bluntly separated from the fibrotic tissue over the MIBS device and from Tenon’s capsule. The fibrotic tissue was then excised, exposing the MIBS device underneath. Fistulation through the device was verified, and a mitomycin C (MMC)-soaked sponge was applied over the bleb area. In some cases, MMC or 5-fluorouracil (5-FU) was injected into the bleb after the conjunctiva was closed. Finally, the conjunctiva was closed with 10.0 nylon sutures. The excised fibrotic tissue was fixed in formalin and sent for histopathological assessment. Written informed consent was obtained at least 24 h before surgery. The date and type of surgeries (XEN or Preserflo implantation, needling, and incisional bleb revision), age, sex, type of glaucoma, pre- and postoperative best-corrected visual acuity, IOP, and number of glaucoma medications taken at different timepoints were documented. This study adhered to the tenets of the Declaration of Helsinki and was approved by the local institutional ethics commission of the Eberhard Karls University, Tuebingen, Germany (489/2022BO2).

Formalin-fixed and paraffin-embedded tissue was sectioned into 5-µm-thick specimens. Hematoxylin and eosin staining was performed for morphological analysis of the specimens. Deparaffinization, rehydration, antigen retrieval, immunostaining, and counterstaining were performed automatically using VENTANA BenchMark GX (Roche Diagnostics, Mannheim, Germany). As primary antibodies, CONFIRM anti-CD68 (KP-1) mouse monoclonal antibody (Roche Diagnostics), actin smooth muscle (1A4) mouse monoclonal antibody (Roche Diagnostics), factor VIII-R Ag. rabbit polyclonal antibody (Roche Diagnostics), CONFIRM anti-vimentin (V9) mouse monoclonal antibody, and podoplanin (D2-40) mouse monoclonal antibody (Roche Diagnostics) were applied. The ultraView Red Detection Kit (Roche Diagnostics) was used for immunostaining and Hematoxylin II and Bluing Reagent (Roche Diagnostics) for counterstaining. For every primary antibody, an appropriate positive control tissue underwent the same procedure.

The incidence of the studied features in the histological slides was documented using a 5-point ordinal scale. The time intervals to the primary surgery were analyzed using the non-parametric Kruskal–Wallis test.

## Results

Thirty-six eyes of 36 patients were included in the study. The median age was 61 years (range, 7–82) and 31% were female. Ten eyes had received an XEN implant, and 26 had received a Preserflo implant. Fourteen patients had primary open-angle glaucoma (POAG), five had pseudoexfoliative glaucoma, two had congenital glaucoma, one patient each had pigment dispersion glaucoma, narrow-angle glaucoma, and ocular hypertension. Twelve patients had secondary glaucoma, including seven with uveitic glaucoma, four with neovascular glaucoma, and one with traumatic glaucoma. Thirteen patients had undergone at least one prior glaucoma surgery, including one Ahmed valve, two trabeculectomies, two trabeculotomies, three XEN implantations, one iStent, and one Cypass implantation, five cyclophotocoagulations, and one cyclocryotherapy.

**Fig. 1:**
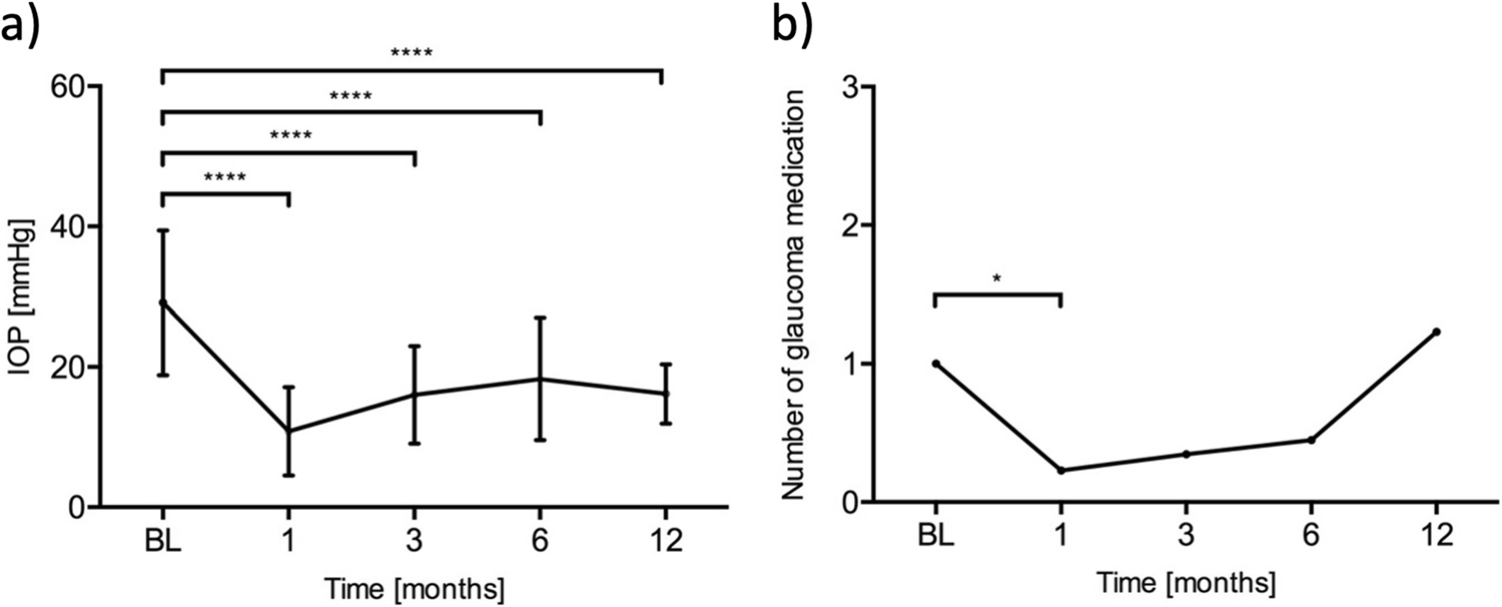
**a** Mean intraocular pressure after bleb revision. Whiskers represent standard deviations. ^****^p < 0.0001. **b** Mean number of glaucoma medications taken after bleb revision. ^*^p < 0.05

The median age at the time of bleb revision was 61 (range = 7–82) years. The median follow-up duration was 6 (range = 1–54) months. Revision surgery was performed at a median of 195 (range = 31–1264) days after primary XEN or Preserflo implantation. The mean IOP before revision surgery was 29.1 (± 10.3) mmHg. It decreased to 10.8 (± 6.3) mmHg (− 63%; *p* < 0.0001, *n* = 35), 16.0 (± 7.0) mmHg (− 45%; *p* < 0.0001, *n* = 26), 18.3 (± 8.7) mmHg (− 37%; *p* < 0.0001, *n* = 20), and 16.2 (± 4.2) mmHg (− 45%; *p* < 0.0001, *n* = 13) after 1, 3, 6, and 12 months, respectively (Fig. 1). The mean number of IOP-lowering medications taken was 1.0 (± 1.7) before revision surgery and 0.2 (± 0.9; *p* = 0.02), 0.4 (± 0.8; *p* = 0.24), 0.5 (± 1.1; *p* = 0.35), and 1.2 (± 1.5; *p* = 0.52) after 1, 3, 6, and 12 months, respectively.

The mean IOP before primary surgery and bleb revision was 30.1 (± 9.2) mmHg and 29.1 (± 10.3) mmHg, respectively (*p* = 0.34). The mean number of IOP-lowering medications taken was significantly higher before primary surgery than before bleb revision: 3.3 (± 1.1) vs. 1.0 (± 1.7; *p* < 0.0001). Fourteen patients developed a temporary hypotony (IOP < 6 mmHg) after revision surgery, which recovered spontaneously after a median of 2 weeks in 13 patients. One patient presented with a flattened anterior chamber and required a viscoelastic injection in the anterior chamber. No choroidal detachment, suprachoroidal bleeding, or other complications involving permanent damage to the eye were seen in any patient.

The mean number of needlings before and after revision surgery was 1.1 (± 0.7) and 0.6 (± 1.0), respectively. The mean time to the first needling was 106 (± 62) days and 144 (± 91) days after primary surgery and revision surgery, respectively (*p* = 0.30).

### Histology

**Fig. 2:**
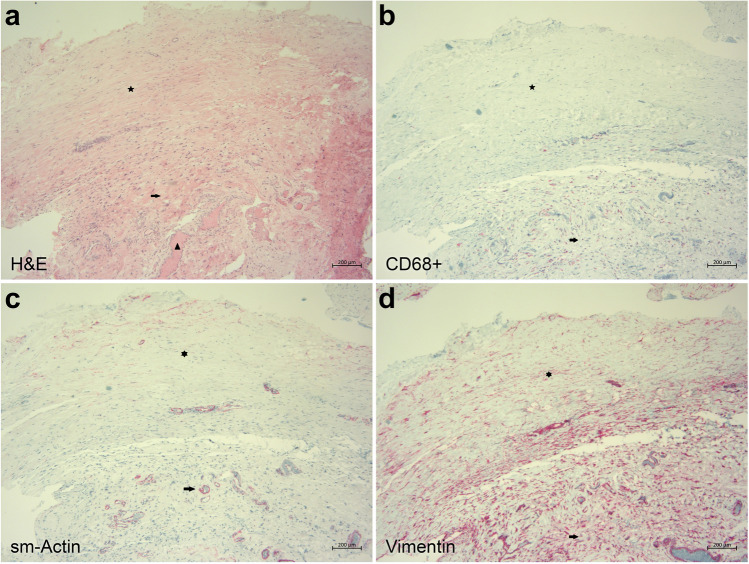
Microphotographs of fibrotic tissue from revision surgery after failed Preserflo MicroShunt implantation. **a** Two different tissue types are seen in this hematoxylin and eosin staining: tight connective tissue (star) and loose connective tissue (arrow). Blood vessels (arrowhead) are predominantly present within the loose connective tissue area. **b** Immunohistochemical staining for macrophages shows that the CD68-positive macrophages (red) are predominantly seen within the loose connective tissue area (arrow) of the specimen in comparison to the tight connective tissue area (star). **c** Immunohistochemical staining for sm-actin (red color) highlights myofibroblasts in the tight connective tissue area (star). In the loose connective tissue area, only myofibroblasts around blood vessels are stained (arrow). **d** Immunohistochemical staining for vimentin (red) demonstrates multiple fibroblasts predominantly in an irregular configuration in the loose connective tissue area (arrow) and a more parallel orientation in the tight connective tissue area (star)

**Fig. 3:**
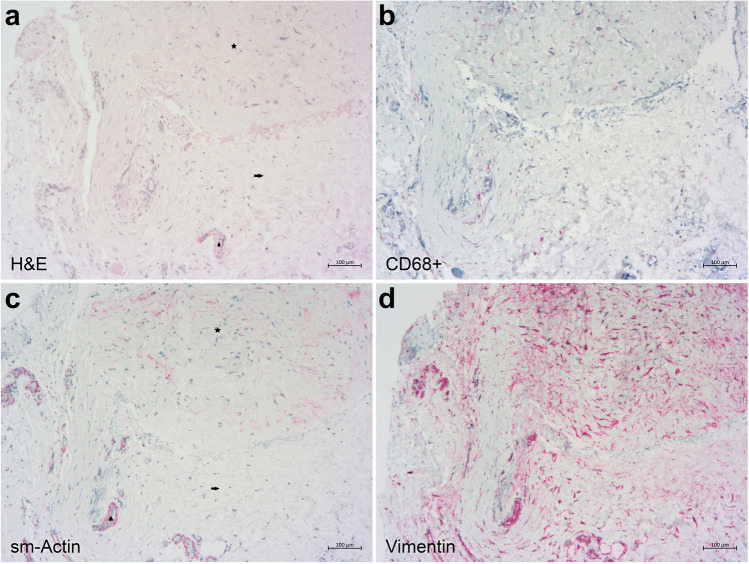
Microphotographs of fibrotic tissue from revision surgery after failed XEN Gel Stent implantation. **a** Predominant tight connective tissue area (star) and predominant loose connective tissue area (arrow) where most blood vessels (arrowhead) are present in this hematoxylin and eosin staining. **b** Immunostaining for CD68 highlights only few macrophages in the fibrotic tissue (red). **c** Immunohistochemical staining for sm-actin (red) demonstrates myofibroblasts within the tight connective tissue area (star). In the loose connective tissue area (arrow), sm-actin is expressed only in the blood vessel-associated myofibroblasts (arrowhead). **d** Immunostaining for vimentin (red) shows multiple fibroblasts in a predominantly irregular arrangement throughout the tissue specimen

Of the 36 samples for histological analysis, 11 were stained only with hematoxylin and eosin. Therefore, no detailed analysis of these samples was possible. The remaining 25 fibrotic tissue samples were analyzed morphologically concerning the formation of tight and loose connective tissue and the presence of inflammation and blood vessels. The samples were also immunohistochemically analyzed with the respective primary antibodies concerning the differentiation of fibroblasts from myofibroblasts and the presence of blood, lymphatic vessels, and macrophages (Figs. 2 and 3).

The baseline demographics, types of glaucoma, and histological results can be found in Table 1.

**Table 1:** Baseline characteristics and results of the histological analysis

Pat ID	Glaucoma	Age	Preserflo/XEN	Time to revision (days)	Previous surgeries	Vimentin	CD68	Factor VIII	Podoplanin	sm-actin	Parallel fibroblasts	Parallel myofibroblasts	Tight connective tissue	Loose connective tissue
1	POWG	73	Preserflo	31		***	*	*	*	(*)	(*)	(*)	*	*
2	POWG	80	Preserflo	129	Cypass, XEN	***	*	*	*	*	*	*	*	*
3	POWG	55	Preserflo	78	CPC	***	*	**	*	**	*	(*)	*	*
4	POWG	76	Preserflo	546	iStent	***	(*)	**	(*)	*	*	/	*	*
5	POWG	64	Preserflo	112		***	*	*	*	*	/	/	*	*
6	POWG	71	Preserflo	138		***	*	*	*	**	/	/	*	*
7	POWG	57	XEN	898		***	(*)	/	(*)	*	/	/	*	/
8	POWG	82	Preserflo	208	CPC	***	*	**	*	*	/	/	*	*
9	POWG	65	Preserflo	156	CPC	***	*	**	(*)	/	*	/	*	*
10	POWG	50	Preserflo	141		***	*	**	*	*	*	*	*	*
11	POWG	57	Preserflo	174		***	*	*	(*)	*	*	*	*	*
12	Uveitic glaucoma	15	Preserflo	308	CPC, CCT	***	(*)	(*)	*	(*)	*	/	*	*
13	Uveitic glaucoma	17	Preserflo	251		***	*	(*)	*	*	*	/	*	*
14	Uveitic glaucoma	52	Preserflo	135		***	*	**	(*)	*	*	/	*	*
15	Uveitic glaucoma	26	Preserflo	399		***	*	*	*	(*)	*	/	*	*
16	Uveitic glaucoma	45	Preserflo	236		***	(*)	*	(*)	(*)	*	/	*	*
17	Uveitic glaucoma	66	XEN	352		***	**	*	(*)	*	/	/	*	*
18	Uveitic glaucoma	71	XEN	481		**	*	(*)	(*)	*	/	/	*	*
19	Secondary glaucoma (neovascular)	38	Preserflo	545	Ahmed	**	(*)	*	*	*	*	(*)	*	*
23	Secondary glaucoma (neovascular)	49	XEN	76		***	*	*	*	*	/	/	*	*
20	Pseudoexfoliative glaucoma	81	Preserflo	181	CPC	***	(*)	*	(*)	**	/	/	*	*
21	Pseudoexfoliative glaucoma	60	Preserflo	45		***	*	*	(*)	*	/	/	*	*
22	Secondary glaucoma (trauma)	31	Preserflo	41		***	**	*	*	**	*	*	*	*
24	Congenital glaucoma	19	Preserflo	40	TO, TE	***	(*)	*	/	/	/	/	/	*
25	Congenital glaucoma	7	XEN	672	TO	***	(*)	**	(*)	*	*	/	*	*

*POWG* primary open-angle glaucoma, *CPC* cyclophotocoagulation, *CCT* cyclocryotherapy, *TO* trabeculotomy, *TE* trabeculectomy

Incidence of the studied features: / not present; (*) singular cells/vessels; * regularly present; ** numerous cells/vessels; *** large quantities of cells/vessels

Tight connective tissue was found in all but one specimen (Figs. 2a and 3a). All specimens contained numerous vimentin-positive activated and enlarged fibroblasts that covered the whole specimen (Figs. 2d and 3d). Sixty percent of the samples (15 of 25) showed fibroblasts in a parallel orientation in a minor part of the probe. In 92% of the samples (23 of 25), sm-actin-positive myofibroblasts were found in a considerably lower quantity than fibroblasts. Approximately 28% (7 of 25) demonstrated myofibroblasts in a parallel orientation (Figs. 2c and 3c). Blood vessels and rare lymphatic vessels were present in all samples. Macrophages were detected in all specimens in similar increased numbers (Figs. 2b and 3b). No pronounced inflammation in the form of lymphocytic or granulocytic infiltration or foreign-body cell reaction was present in any samples. There was no observed difference between XEN or Preserflo samples and the incidence of the assessed histological changes was not associated with the time interval between primary surgery and bleb revision surgery.

A subgroup analysis was also conducted to investigate histological differences between patients with uveitic secondary glaucoma (*n* = 7) and POAG (*n* = 11). No significant differences were found between the two groups in any of the investigated features.

Furthermore, the two patients diagnosed with congenital glaucoma did not exhibit any significant differences compared to cases of other types of glaucoma.

## Discussion

Patients with advanced stages of glaucoma remain challenging to treat, especially when filtering surgeries fail owing to bleb fibrosis. Despite intensive research on bleb fibrosis, most studies have focused on trabeculectomy, and few have involved patients with XEN or Preserflo implants. In this regard, the data presented in this study may contribute to a better understanding of bleb fibrosis after MIBS with the XEN or Preserflo.

In our study, almost all samples demonstrated characteristic fibrotic features such as activated and enlarged fibroblasts in either parallel or irregular orientations in failed blebs after implantation of the XEN or Preserflo. Multimodal imaging with confocal microscopy and OCT has previously demonstrated a significant increase in reflectivity of failed XEN blebs [16], likely attributable to the presence of increased fibrosis, as confirmed in our histopathological analysis. These results align with previous reports on failed trabeculectomy eyes, in which a similar increase in the reflectivity of the bleb and a high density of collagen connective tissue were found [17, 18].

In general, three factors are believed to be important in the development of filtering bleb fibrosis: first, the trauma of the surgery, which initiates the wound healing process and inflammation; second, the new aqueous humor channel, which brings the aqueous humor into direct contact with the wound; and third, the surface of the implanted material.

Regarding postoperative inflammation, the presence of many lymphocytes and neutrophil granulocytes is expected, but in this study, we observed large numbers of macrophages only and no other inflammatory cells. In addition, the fibrotic features of the blebs were not dependent on the time after primary surgery. Taken together, these observations indicated that postoperative inflammation was not the major cause of fibrosis in our patients. Factors related to the aqueous humor might play a more important role by activating subconjunctival macrophages and fibroblasts, leading to bleb fibrosis.

It has long been discussed that the aqueous humor is involved in processes that result in post-trabeculectomy bleb fibrosis and, finally, surgical failure [19, 20]. Cytokines present in the aqueous humor such as TGF-β, PDGF, VEGF, and TNF-α are important factors in postoperative wound healing. Their role in bleb fibrosis has been extensively studied. The concentrations of these cytokines are largely increased in the vitreous humor of patients with glaucoma [21–23]. In particular, TGF-β seems to induce the differentiation of fibroblasts to α-SMA-expressing myofibroblasts, which can modulate the extracellular matrix and exhibit contractile properties, contributing to bleb fibrosis and failure. Accordingly, we found sm-actin-positive myofibroblasts in almost all samples. Interestingly, different types of glaucoma seem to have varying concentrations of cytokines in the vitreous [24]. However, our analysis of patients with POAG and uveitic glaucoma showed no differences in any of the histological parameters analyzed. This may be due to the small sample size, but it is also consistent with the finding that trabeculectomy in secondary uveitic glaucoma produces comparable results to those in patients with POAG [25, 26].

The fibrotic response to MIBS has also been previously studied in animals. One study investigated the fibrotic response to the Preserflo implant in rabbits and found polymorphonuclear leukocytes, foreign body giant cells, and many myofibroblasts located mostly in the fibrotic bleb wall 40 days after surgery [27]. In contrast, another study analyzed rabbit eyes 100 days after Preserflo implantation and found no myofibroblasts [28]. The latter finding mostly agrees with that of our study, although a comparison between human and rabbit eyes should be undertaken with caution. We did not observe any giant multinucleated cells in either the XEN- or Preserflo-implanted eyes but noted singular myofibroblasts in 23 of 25 samples and parallel-oriented myofibroblasts in 7 of 25 samples. Similarly, the XEN implant has not been reported to cause pronounced immune reactions in rabbits and dogs [29].

The stent surface is another factor influencing postoperative bleb fibrosis. Accordingly, efforts have been made to use innovative materials to reduce bleb fibrosis. For example, nanofiber-based materials mimic the natural extracellular matrix better than the flat surfaces of XEN implants or the silicon tube of Baerveldt glaucoma implants [30]. A recent study showed that this new material minimized biomaterial-associated bleb fibrosis in rabbits and could extend the lifespan of a filtrating stent [30]. Thus, a combination of surface material modification and longer-term cytokine milieus regulation is likely crucial in ensuring the long-term success of glaucoma implants and in inhibiting bleb fibrosis.

Numerous pre-, intra-, and postoperative interventions have been studied over the past decades in an attempt to reduce the risk of bleb fibrosis and failure [31, 32]. The use of antimetabolites such as MMC and 5-FU is now well established in glaucoma surgery and can improve long-term success, although side effects along with non-cell-specific cytotoxic effects must be acknowledged [33]. Nevertheless, intraoperative administration of antimetabolites can have only a temporary influence on the local environment, whereas prolonged effects might be necessary for long-term success. A previous histological analysis of failed trabeculectomies showed that eyes operated without MMC presented with parallel-oriented fibroblasts, whereas eyes operated with MMC had only few fibroblasts, with neither contractile fibers nor a particular orientation [19]. This finding contrasts with our data that nearly all samples contained numerous fibroblasts, and 60% presented with parallel-oriented fibroblasts, indicating strong bleb fibrosis, even though all patients received MMC during primary surgery. Additionally, the majority of our samples stained positive for singular sm-actin-positive myofibroblasts. In the abovementioned previous histological analysis, no association was found between histological features and time after surgery, agreeing with our findings.

A limitation of our study is that we included patients with different types of glaucoma and varying numbers of previous surgeries, which could have influenced the development of bleb fibrosis. However, this approach allowed us to compare the development of bleb fibrosis under different conditions and might enable better generalizability to clinical practice. It is also important to keep in mind that all patients underwent surgery due to a clinically significant bleb fibrosis. Therefore, it is not surprising that the histological results do not differ significantly, despite the patients having different types of glaucoma.

In conclusion, our study provides important insights into the biocompatibility of the XEN and Preserflo implants in humans. The histological analyses revealed consistent histological patterns across a diverse patient population, suggesting that the findings may be generalizable across a wide range of patients. Most importantly, no patient showed signs of a pronounced immune or foreign-body reaction, underlining the value of the XEN and Preserflo implants in the management of treatment-refractory glaucoma.

## Data Availability

Due to ethical and legal reasons, data is not publicly available.

## References

[1] Nouri-Mahdavi K, Brigatti L, Weitzman M, Caprioli J (1995) Outcomes of trabeculectomy for primary open-angle glaucoma. Ophthalmology 102:1760–1769. 10.1016/s0161-6420(95)30796-8 9098275 10.1016/s0161-6420(95)30796-8

[2] Cairns JE (1968) Trabeculectomy. Preliminary report of a new method. Am J Ophthalmol 66:673–679 4891876 10.1016/0002-9394(68)91288-9

[3] Kirwan JF, Lockwood AJ, Shah P, Macleod A, Broadway DC, King AJ, McNaught AI, Agrawal P, Trabeculectomy Outcomes Group Audit Study G (2013) Trabeculectomy in the 21st century: a multicenter analysis. Ophthalmology 120:2532–2539. 10.1016/j.ophtha.2013.07.049 24070811 10.1016/j.ophtha.2013.07.049

[4] Lavia C, Dallorto L, Maule M, Ceccarelli M, Fea AM (2017) Minimally-invasive glaucoma surgeries (MIGS) for open angle glaucoma: a systematic review and meta-analysis. PLoS ONE 12:e0183142. 10.1371/journal.pone.0183142 28850575 10.1371/journal.pone.0183142 PMC5574616

[5] Fea AM, Bron AM, Economou MA, Laffi G, Martini E, Figus M, Oddone F (2020) European study of the efficacy of a cross-linked gel stent for the treatment of glaucoma. J Cataract Refract Surg 46:441–450. 10.1097/j.jcrs.0000000000000065 32142041 10.1097/j.jcrs.0000000000000065

[6] Schargus M, Theilig T, Rehak M, Busch C, Bormann C, Unterlauft JD (2020) Outcome of a single XEN microstent implant for glaucoma patients with different types of glaucoma. BMC Ophthalmol 20:490. 10.1186/s12886-020-01764-8 33334311 10.1186/s12886-020-01764-8 PMC7745382

[7] Reitsamer H, Sng C, Vera V, Lenzhofer M, Barton K, Stalmans I, Apex Study G (2019) Two-year results of a multicenter study of the ab interno gelatin implant in medically uncontrolled primary open-angle glaucoma. Graefes Arch Clin Exp Ophthalmol 257:983–996. 10.1007/s00417-019-04251-z 30758653 10.1007/s00417-019-04251-z

[8] Faber H, Guggenberger V, Voykov B (2022) XEN45 Gelstent implantation in the treatment of glaucoma secondary to fuchs uveitis syndrome. Ocul Immunol Inflamm 30:1678–1685. 10.1080/09273948.2021.1934035 34124988 10.1080/09273948.2021.1934035

[9] Beckers HJM, Aptel F, Webers CAB, Bluwol E, Martinez-de-la-Casa JM, Garcia-Feijoo J, Lachkar Y, Mendez-Hernandez CD, Riss I, Shao H, Pinchuk L, Angeles R, Sadruddin O, Shaarawy TM (2022) Safety and effectiveness of the PRESERFLO(R) MicroShunt in primary open-angle glaucoma: results from a 2-year multicenter study. Ophthalmol Glaucoma 5:195–209. 10.1016/j.ogla.2021.07.008 34329772 10.1016/j.ogla.2021.07.008

[10] Schlenker MB, Durr GM, Michaelov E, Ahmed IIK (2020) Intermediate outcomes of a novel standalone ab externo SIBS microshunt with mitomycin C. Am J Ophthalmol 215:141–153. 10.1016/j.ajo.2020.02.020 32173344 10.1016/j.ajo.2020.02.020

[11] Fea AM, Laffi GL, Martini E, Economou MA, Caselgrandi P, Sacchi M, Au L (2022) Effectiveness of MicroShunt in patients with primary open-angle and pseudoexfoliative glaucoma: a retrospective European multicenter study. Ophthalmol Glaucoma 5:210–218. 10.1016/j.ogla.2021.08.005 34478904 10.1016/j.ogla.2021.08.005

[12] Jamke M, Herber R, Haase MA, Jasper CS, Pillunat LE, Pillunat KR (2023) PRESERFLO MicroShunt versus trabeculectomy: 1-year results on efficacy and safety. Graefes Arch Clin Exp Ophthalmol 261:2901–2915. 10.1007/s00417-023-06075-4 37133501 10.1007/s00417-023-06075-4 PMC10155172

[13] Panarelli JF, Moster MR, Garcia-Feijoo J, Flowers BE, Baker ND, Barnebey HS, Grover DS, Khatana AK, Lee B, Nguyen T, Stiles MC, Sadruddin O, Khaw PT, Group INNS (2023) Ab-externo microshunt versus trabeculectomy in primary open-angle glaucoma: two-year results from a randomized, multicenter study. Ophthalmology. 10.1016/j.ophtha.2023.09.023 37769852 10.1016/j.ophtha.2023.09.023

[14] Cabourne E, Clarke JC, Schlottmann PG, Evans JR (2015) Mitomycin C versus 5-Fluorouracil for wound healing in glaucoma surgery. Cochrane Database Syst Rev 2015: CD006259. 10.1002/14651858.CD006259.pub2 10.1002/14651858.CD006259.pub2 PMC8763343 26545176

[15] Mansouri K, Bravetti GE, Gillmann K, Rao HL, Ch’ng TW, Mermoud A (2019) Two-year outcomes of XEN gel stent surgery in patients with open-angle glaucoma. Ophthalmol Glaucoma 2:309–318. 10.1016/j.ogla.2019.03.011 32672673 10.1016/j.ogla.2019.03.011

[16] Fea AM, Spinetta R, Cannizzo PML, Consolandi G, Lavia C, Aragno V, Germinetti F, Rolle T (2017) Evaluation of bleb morphology and reduction in IOP and glaucoma medication following implantation of a novel gel stent. J Ophthalmol 2017:9364910. 10.1155/2017/9364910 28751986 10.1155/2017/9364910 PMC5511657

[17] Addicks EM, Quigley HA, Green WR, Robin AL (1983) Histologic characteristics of filtering blebs in glaucomatous eyes. Arch Ophthalmol 101:795–798. 10.1001/archopht.1983.01040010795021 6847472 10.1001/archopht.1983.01040010795021

[18] Ciancaglini M, Carpineto P, Agnifili L, Nubile M, Lanzini M, Fasanella V, Mastropasqua L (2008) Filtering bleb functionality: a clinical, anterior segment optical coherence tomography and in vivo confocal microscopy study. J Glaucoma 17:308–317. 10.1097/IJG.0b013e31815c3a19 18552617 10.1097/IJG.0b013e31815c3a19

[19] Mietz H, Arnold G, Kirchhof B, Diestelhorst M, Krieglstein GK (1996) Histopathology of episcleral fibrosis after trabeculectomy with and without mitomycin C. Graefes Arch Clin Exp Ophthalmol 234:364–368. 10.1007/BF00190712 8738702 10.1007/BF00190712

[20] Teng CC, Chi HH, Katzin HM (1959) Histology and mechanism of filtering operations. Am J Ophthalmol 47:16–33. 10.1016/s0002-9394(14)78135-8 13617347 10.1016/s0002-9394(14)78135-8

[21] Ochiai Y, Ochiai H (2002) Higher concentration of transforming growth factor-beta in aqueous humor of glaucomatous eyes and diabetic eyes. Jpn J Ophthalmol 46:249–253. 10.1016/s0021-5155(01)00523-8 12063033 10.1016/s0021-5155(01)00523-8

[22] Agarwal P, Daher AM, Agarwal R (2015) Aqueous humor TGF-beta2 levels in patients with open-angle glaucoma: a meta-analysis. Mol Vis 21:612–620 26019480 PMC4445076

[23] Chen H, Zheng G, Chen H, Li L, Xu Z, Xu L (2022) Evaluations of aqueous humor protein markers in different types of glaucoma. Medicine (Baltimore) 101:e31048. 10.1097/MD.0000000000031048 36254076 10.1097/MD.0000000000031048 PMC9575751

[24] Inatani M, Tanihara H, Katsuta H, Honjo M, Kido N, Honda Y (2001) Transforming growth factor-beta 2 levels in aqueous humor of glaucomatous eyes. Graefes Arch Clin Exp Ophthalmol 239:109–113. 10.1007/s004170000241 11372538 10.1007/s004170000241

[25] Kanaya R, Kijima R, Shinmei Y, Shinkai A, Ohguchi T, Namba K, Chin S, Ishida S (2021) Surgical outcomes of trabeculectomy in uveitic glaucoma: a long-term, single-center, retrospective case-control study. J Ophthalmol 2021:5550776. 10.1155/2021/5550776 34094594 10.1155/2021/5550776 PMC8163556

[26] Kaburaki T, Koshino T, Kawashima H, Numaga J, Tomidokoro A, Shirato S, Araie M (2009) Initial trabeculectomy with mitomycin C in eyes with uveitic glaucoma with inactive uveitis. Eye (Lond) 23:1509–1517. 10.1038/eye.2009.117-cme 19521438 10.1038/eye.2009.117-cme

[27] van Mechelen RJS, Wolters JEJ, Herfs M, Bertens CJF, Gijbels M, Pinchuk L, Gorgels T, Beckers HJM (2022) Wound healing response after bleb-forming glaucoma surgery with a SIBS microshunt in rabbits. Transl Vis Sci Technol 11:29. 10.1167/tvst.11.8.29 36018582 10.1167/tvst.11.8.29 PMC9428362

[28] Acosta AC, Espana EM, Yamamoto H, Davis S, Pinchuk L, Weber BA, Orozco M, Dubovy S, Fantes F, Parel JM (2006) A newly designed glaucoma drainage implant made of poly(styrene-b-isobutylene-b-styrene): biocompatibility and function in normal rabbit eyes. Arch Ophthalmol 124:1742–1749. 10.1001/archopht.124.12.1742 17159034 10.1001/archopht.124.12.1742

[29] Shute TS, Dietrich UM, Baker JF, Carmichael KP, Wustenberg W, Ahmed II, Sheybani A (2016) Biocompatibility of a novel microfistula implant in nonprimate mammals for the surgical treatment of glaucoma. Invest Ophthalmol Vis Sci 57:3594–3600. 10.1167/iovs.16-19453 27391549 10.1167/iovs.16-19453

[30] Josyula A, Mozzer A, Szeto J, Ha Y, Richmond N, Chung SW, Rompicharla SVK, Narayan J, Ramesh S, Hanes J, Ensign L, Parikh K, Pitha I (2023) Nanofiber-based glaucoma drainage implant improves surgical outcomes by modulating fibroblast behavior. Bioeng Transl Med 8:e10487. 10.1002/btm2.10487 37206200 10.1002/btm2.10487 PMC10189467

[31] Theilig T, Papadimitriou M, Albaba G, Meller D, Hasan SM (2023) Results of open bleb revision as management of primary bleb failure following XEN 45 gel stent and Preserflo Microshunt. Graefes Arch Clin Exp Ophthalmol. 10.1007/s00417-023-06152-8 37410178 10.1007/s00417-023-06152-8 PMC10587268

[32] Schlunck G, Meyer-ter-Vehn T, Klink T, Grehn F (2016) Conjunctival fibrosis following filtering glaucoma surgery. Exp Eye Res 142:76–82. 10.1016/j.exer.2015.03.021 26675404 10.1016/j.exer.2015.03.021

[33] Wilkins M, Indar A, Wormald R (2005) Intra-operative mitomycin C for glaucoma surgery. Cochrane Database Syst Rev 2005: CD002897. 10.1002/14651858.CD002897.pub2 10.1002/14651858.CD002897.pub2 PMC8406713 16235305

